# Epidemiological and economic burden of metabolic syndrome and its consequences in patients with hypertension in Germany, Spain and Italy; a prevalence-based model

**DOI:** 10.1186/1471-2458-10-529

**Published:** 2010-09-02

**Authors:** Jürgen Scholze, Eduardo Alegria, Claudio Ferri, Sue Langham, Warren Stevens, David Jeffries, Kerstin Uhl-Hochgraeber

**Affiliations:** 1 Department of Medicine, Outpatient Clinic, CCM, Charite-Universitatsmedizin Berlin, Luisenstrasse 11-13, 10117 Berlin, Germany; 2 Servicio de Cardiología, Policlínica Gipuzkoa, San Sebastián, Spain; 3 Professor of Internal Medicine, Department of Internal Medicine and Public Health, Division of Internal Medicine, Hypertension & Cardiovascular Prevention Center, University of L'Aquila, San Salvatore Hospital, L'Aquila, Italy; 4 Independent Health Economist, Manchester, UK; 5 Independent Health Economist, Boston, USA; 6 Head of Statistics and Data Management, MRC Tropical Disease Research Unit, Banjul, Gambia; 7 Bayer Schering Pharma AG, Global Health Economics & Outcomes Research, Berlin, Germany

## Abstract

**Background:**

The presence of metabolic syndrome in patients with hypertension significantly increases the risk of cardiovascular disease, type 2 diabetes and mortality. Our aim is to estimate the epidemiological and economic burden to the health service of metabolic syndrome in patients with hypertension in three European countries in 2008 and 2020.

**Methods:**

An age, sex and risk group structured prevalence based cost of illness model was developed using the United States Adult Treatment Panel III of the National Cholesterol Education Program criteria to define metabolic syndrome. Data sources included published information and public use databases on disease prevalence, incidence of cardiovascular events, prevalence of type 2 diabetes, treatment patterns and cost of management in Germany, Spain and Italy.

**Results:**

The prevalence of hypertension with metabolic syndrome in the general population of Germany, Spain and Italy was 36%, 11% and 10% respectively. In subjects with hypertension 61%, 22% and 21% also had metabolic syndrome. Incident cardiovascular events and attributable mortality were around two fold higher in subjects with metabolic syndrome and prevalence of type 2 diabetes was around six-fold higher. The economic burden to the health service of metabolic syndrome in patients with hypertension was been estimated at €24,427, €1,900 and €4,877 million in Germany, Spain and Italy and forecast to rise by 59%, 179% and 157% respectively by 2020. The largest components of costs included the management of prevalent type 2 diabetes and incident cardiovascular events. Mean annual costs per hypertensive patient were around three-fold higher in subjects with metabolic syndrome compared to those without and rose incrementally with the additional number of metabolic syndrome components present.

**Conclusion:**

The presence of metabolic syndrome in patients with hypertension significantly inflates economic burden and costs are likely to increase in the future due to an aging population and an increase in the prevalence of components of metabolic syndrome.

## Background

High blood pressure is a leading cause of death and disability causing 13.5% of the world's premature death and 6% of its disability. Half of all strokes and ischemic heart disease can be attributed to high blood pressure[1]. This situation shows no sign of abating. Obesity, a major risk factor for hypertension, has reached pandemic proportions [2] and research shows that around two thirds of the prevalence of hypertension is directly attributable to obesity[3]. Along with obesity, other independent cardiometabolic abnormalities, such as dyslipidaemia, hypertriglyceridaemia and glucose metabolism disturbances have also been found to cluster together. Such groupings of risk factors for cardiovascular disease in the same hypertensive individual occur together more often than would be expected by chance alone, giving rise to a clinical entity that has been termed metabolic syndrome (MetS)[4]. The syndrome has been shown to significantly increase the risk of cardiovascular disease, type 2 diabetes and mortality[5-8]. In patients with hypertension it doubles the relative risk of cardiovascular disease[9-11] and triples the relative risk of type 2 diabetes[12]. Moreover, the risk increases with the number of MetS components present[10,13]. As there are 16 different conceivable combinations of risk factors that could be diagnosed as MetS not all can be weighted equally in terms of their impact on risk and for some combinations this increase in risk is controversial. However, what is known for sure is that the coexistence of hypertension disproportionately increases the risk of cardiovascular disease[14].

However, there is much debate as to whether MetS should be treated as a clinical entity in general practice or whether physicians should concentrate on treating individual risk factors. The primary purpose for diagnosing MetS in general practice is to identify patients who are at high long-term risk of developing cardiovascular disease and type 2 diabetes and who require lifestyle and/or pharmacological therapies to reduce this risk. Recent European guidelines on the management of patients with arterial hypertension consider those with hypertension and MetS as a special condition suggesting a different therapeutic approach compared to patients with hypertension alone[15]. Such recommendations are based on a number of evidence-based observations. In a position statement the European Society of Hypertension pointed out that in patients with hypertension and MetS the overall cardiovascular risk may be greater than the sum of its identifiable components and that MetS components are often defined by values lower than those defined in various individual risk factor guidelines which may lead to many patients with high cardiovascular risk not being identified[16]. They also point out that MetS risk factors are relatively easily identified in clinical practice.

The clinical utility of diagnosing MetS in general practice has been hampered by the inconsistency of the diagnostic criteria available. Some definitions appear to be better than others at identifying high risk patients in Europe. In studies using both the International Diabetes Federation criteria and those developed by the United States Adult Treatment Panel III of the National Cholesterol Education Program (ATP III) a much higher prevalence of MetS was identified by the International Diabetes Federation criteria[17,18] but these criteria led to much lower predictive power for coronary events than the ATP III criteria[19]. This suggests that currently the ATP III criteria are the most appropriate for European populations given that excessive over-diagnosing of the syndrome would subject the health service to unnecessary budget pressures.

Relatively little is known about the epidemiological burden of MetS in patients with hypertension in the general population in Europe. Using ATP III criteria, European population studies suggest that the prevalence is around 8% to 13%[20-22]. The reported proportion of hypertensive patients that have MetS is wide-ranging. Population-based studies suggest the proportion is around 20% to 40%[20-23]. Primary care studies report that a fifth and up to just over a half of hypertension patients can be diagnosed with MetS[13,17,24-27].

No studies have assessed the economic burden of MetS in patients with hypertension. In order to fill this gap this study aims to model the health care costs of hypertension in three European countries (Germany, Spain and Italy) in 2008 and 2020 and to assess how the consequences of MetS in terms of associated type 2 diabetes and the increase in cardiovascular events impacts on this economic burden.

## Methods

### Overview

This study uses a prevalence-based approach that combined the demographics of the population with hypertension and MetS prevalence rates, incidence of cardiovascular disease, prevalence of type 2 diabetes and healthcare costs into a cost of hypertension and MetS model. A comprehensive literature review was conducted to identify inputs and data sources included national surveys, IMS Health Pharmaceuticals Sales data and published information. The model and the assumptions used have been described in detail elsewhere[28].

**Figure 1:**
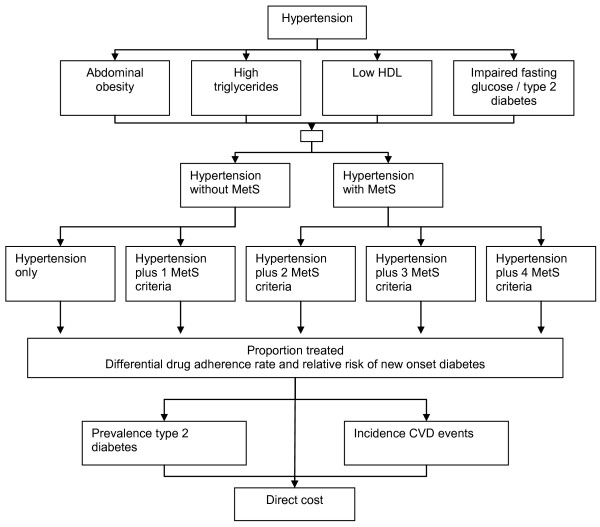
**Schematic representation of the burden of illness model**.

Briefly we developed a prevalence-based model from the perspective of the health service (Figure 1). MetS was defined according to modified ATP III criteria, where an individual is defined as having MetS if they have three out of five diagnostic criteria: Abdominal obesity (waist; males > 102 cm, females > 88 cm), hypertension (≥140/90 mm Hg), low high-density lipoprotein (HDL) cholesterol (males <40 mg/dL, females <50 mg/dL), high triglycerides (≥ 150 mg/dL) and impaired fasting glucose (≥ 110 mg/dL or known diabetes)[29]. We modified the criteria in relation to blood pressure from ≥135/85 mm Hg to ≥140/90 mm Hg for two reasons; first all epidemiological publications and databases used to develop the model defined hypertension as blood pressure of ≥140/90 mm Hg and second, because current European and U.S. guidelines use this definition as the treatment threshold for antihypertensive medication[15,30].

The model considered all patients in Germany, Spain and Italy over the age of 20 years. Two sets of risk groups were defined, totalling seven risk categories. Risk was stratified first into two categories - those patients with hypertension and MetS and those without - where MetS was indicated by the hypertensive individual having at least two other ATP III criteria, and second into five risk categories - those patients with hypertension only, hypertension plus one other ATP III criteria, hypertension plus two other, hypertension plus three other and hypertension plus four other ATP III criteria (Figure 1). In order to breakdown the hypertension population data into these risk groups we analysed the United States National Health and Nutrition Examination Survey (NHANES) public use database (2000) to assess the conditional structure for the five components of MetS and applied this conditional structure to country-specific prevalence data.

### Data sources

Prevalence data were sourced from a national survey in Germany[31] and published epidemiological studies in Spain[17,32] and Italy[22,33]. In order to estimate the future prevalence up to 2020 compound annual growth rates (CAGRs) were derived from the literature[34] or a multiyear evaluation of the NHANES public use database.

Each risk group was divided into those who receive antihypertensive drug treatment and those who don't. For the treated group the proportion and cost of hypertensive subjects treated with each antihypertensive drug class (monotherapy and combination therapy for angiotensin II receptor blockers (ARBs), angiotensin converting enzyme (ACE) inhibitors, calcium channel blockers, diuretics and beta-blockers) were derived from an analysis of IMS Health pharmaceutical national sales data for all main antihypertensive drug classes prescribed in 2007 to patients with an ICD-10 code for hypertension. Treated patients were also assumed to have associated costs for primary care visits to the clinic for check-ups, tests and assessment of hypertensive medication which were derived from country specific publications[35-37]. For the treated group the model took into account the differential adherence rate[38] and relative risk of treatment-related new onset diabetes between the drug classes[39].

For both treated and untreated patients the incidence of cardiovascular events and prevalence of type 2 diabetes and their associated costs were estimated. Cardiovascular events included acute myocardial infarction, congestive heart failure, unstable angina, stroke and death. Incidence and costs of events were derived from the literature[11,13,40-45]. The prevalence of type 2 diabetes in the hypertensive population was derived from a European national survey[31] and costs derived from published country-specific data[46].

The cost data in the model takes the viewpoint of the health service and includes hypertension drug costs, management of hypertension in primary care and costs associated with the treatment and management of cardiovascular events and type 2 diabetes. The cost of each of these elements was calculated by multiplying the quantity of the resource used or the number of events with the unit price. All costs were inflated to 2008 prices and presented in Euros.

### Analyses

The burden of illness model was created in Access. The Access model generated epidemiological numbers and costs which were then exported to excel for analysis. The model was quality controlled by cross-checking epidemiological and cost data with other published reports.

To address uncertainty around the mean in the overall cost of illness estimate, we conducted a univariate sensitivity analysis, where one factor at a time was varied while keeping all other factors constant at their base-case value. We assessed the impact on cost-of-illness of; only including incident rather than prevalent type 2 diabetes, varying the continuation rate, drug cost, cardiovascular cost and type 2 diabetes cost by plus and minus 20%; and applying a discount rate of 3% to future costs.

## Results

### Prevalence and risk groups

**Figure 2:**
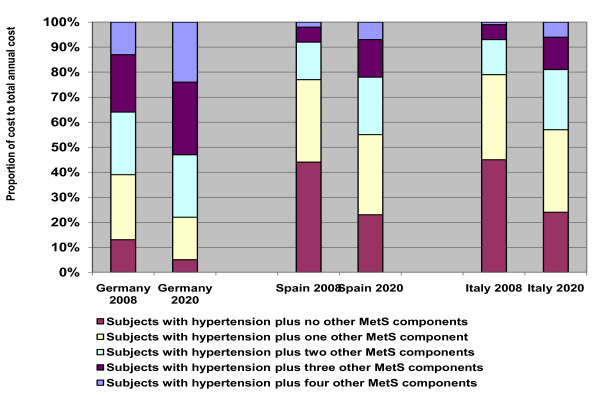
**Proportion of the hypertensive population > 20 years within each risk group; 2008-2020**.

The prevalence of hypertension with MetS in the general population of Germany, Spain and Italy was 36%, 11% and 10% respectively in 2008. In Germany of all subjects with hypertension over half (61%) complied with the ATP III criteria for MetS. In Spain and Italy this proportion was much lower with 22% and 21% respectively. By 2020 the proportion of the hypertensive population with MetS was forecast to increase to 78%, 45% and 43% in Germany, Spain and Italy respectively giving a prevalence in the general population of hypertension with MetS of 48% in Germany and 22% in Spain and Italy. Germany had a higher proportion of hypertensive subjects with three or four other components of MetS (36%) compared to Spain (8%) and Italy (7%) (figure 2).

**Figure 3:**
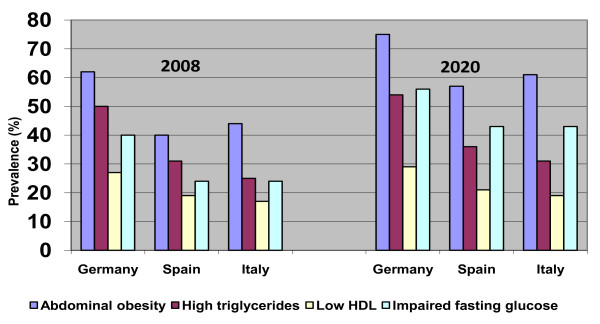
**Prevalence of individual components of MetS in patients with hypertension; 2008 and 2020**.

Figure 3 outlines the prevalence of each individual MetS component in patients with hypertension in 2008 and 2020. The most prevalent component was abdominal obesity which reached a prevalence of 62%, 40% and 44% in hypertensive patients in Germany, Spain and Italy respectively in 2008. This was followed by high tryiglycerides, and then impaired fasting glucose. All MetS components were forecast to rise with the prevalence of abdominal obesity and impaired fasting glucose increasing the most.

### Consequences of disease

The incidence of cardiovascular disease and mortality was around two-fold higher in hypertensive subjects with MetS compared to those without in all three countries. The prevalence of type 2 diabetes was around six fold higher in subjects with MetS (table 1). The incidence of cardiovascular disease and mortality and the prevalence of type 2 diabetes increased incrementally with each additional component of MetS. The total number of cardiovascular events, deaths and cases of type 2 diabetes are forecast to rise over 100% in Spain and Italy respectively between 2008 and 2020 and by around 50% in Germany (table 1).

**Table 1:** Annual number and event rate per 1,000 hypertensive patients of incident cases of cardiovascular disease, mortality and prevalent cases of type 2 diabetes; 2008 and 2020.

	Germany		Spain		Italy	
	**No. events**	Event rate*	**No. events**	Event rate*	**No. events**	Event rate*

**Annual incidence of cardiovascular events**						

**2008**						
MetS†	583,500	27	74,600	25	103,300	24
No MetS‡	188,500	14	134,000	13	202,500	13
**2020**						
MetS†	889,500	29	208,300	27	267,800	25
No MetS‡	124,400	14	132,200	14	189,900	13

**Annual incidence of mortality**						

**2008**						
MetS†	64,000	3.00	14,800	2.74	11,300	2.64
No MetS‡	20,600	1.49	8,200	1.45	22,400	1.40
**2020**						
MetS†	97,800	3.17	22,800	2.90	29,300	2.78
No MetS‡	13,600	1.52	14,500	1.50	20,800	1.50

**Annual prevalence of type 2 diabetes**						

**2008**						
MetS†	5,287,600	248	834,500	281	1,313,300	308
No MetS‡	627,300	45	515,100	51	823,700	52
**2020**						
MetS†	8,676,100	281	2,364,500	301	3,390,200	322
No MetS‡	510,300	57	703,400	73	1,021,600	71

* Event rate per 1000, † hypertension patients with MetS, ‡ hypertension patients without MetS.

### Costs

Table 2 details the total annual cost-of-illness of hypertension with and without MetS in 2008 and 2020 in Germany, Spain and Italy. Total annual costs of hypertension with MetS amounted to €24,427, €1,909 and €4,877 million respectively in Germany, Spain and Italy in 2008. These costs represented 82%, 42% and 45% of the total annual costs of hypertension. By 2020, keeping costs set at 2008 prices, these annual costs of hypertension with MetS were forecast to rise by 59%, 179%, 157% in Germany, Spain and Italy respectively (table 2).

**Table 2:** Annual cost-of-illness (Euros, millions) of hypertension and the proportion of costs attributable to MetS; 2008 and 2020.

	Annual costs				
	**Drug^1^**	**Physician^2^**	**CVD^3^**	**Type 2 diabetes^4^**	**Total**

**2008**					

**Germany**					
With MetS†	628	1,952	5,265	16,582	**24,427**
Without MetS‡	407	1,264	1,703	1,967	**5,341**

**Spain**					
With MetS	116	126	699	968	**1,909**
Without MetS	397	432	1,256	597	**2,682**

**Italy**					
With MetS	258	330	817	3,472	**4,877**
Without MetS	958	1,222	1,599	2,178	**5,957**

**2020**					

**Germany**					
With MetS†	901	2,820	8,026	27,208	**38,955**
Without MetS‡	270	817	1,124	1,600	**3,811**

**Spain**					
With MetS	301	333	1,952	2,743	**5,329**
Without MetS	384	412	1,240	816	**2,852**

**Italy**					
With MetS	629	813	2,117	8,964	**12,523**
Without MetS	877	1,109	1,500	2,701	**6,187**

CVD-cardiovascular disease.

† hypertension patients with MetS, ‡ hypertension patients without MetS.

^1^Drug costs relate to antihypertensive treatments only, ^2^physician costs relate to visits to the clinic as a result of hypertension for check-ups and assessment of antihypertensive medication, ^3^CVD costs relate to ambulatory and hospitalisation costs for the management of each event, ^4^type 2 diabetes costs relate to the management of each patients and the costs of non-CVD related complications.

**Figure 4:**
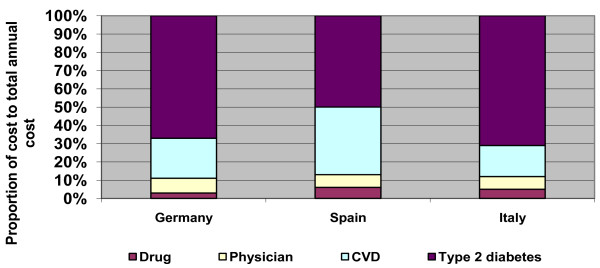
**Proportion of drug, physician, cardiovascular and type 2 diabetes costs to total annual costs for patients with hypertension and MetS; 2008**.

The largest component of the total annual cost of hypertensive patients with MetS was the treatment and management of the consequence of disease rather than the management of hypertension itself including physician and drug costs. Prevalent type 2 diabetes and incident cardiovascular disease constituted 67% and 22% of total costs respectively in Germany in 2008 (figure 4). In Spain these proportions were 50% and 37% and in Italy were 71% and 17%. Hypertension drug costs and physician costs relating to the management of hypertension constituted the smallest proportion of total costs in all countries (figure 4).

**Figure 5:**
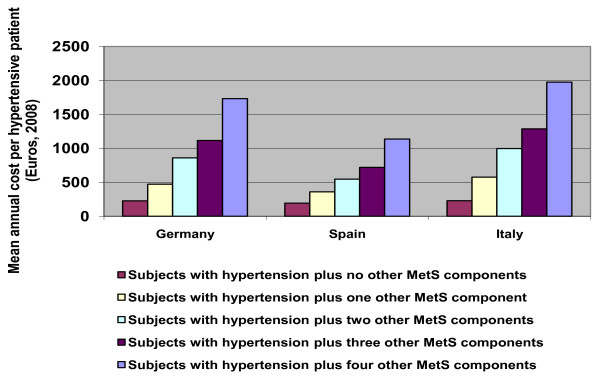
**Mean annual costs (Euros) per hypertensive patient according to the number of components of MetS; 2008**.

Mean annual costs per hypertensive patient were around three-fold higher in subjects with MetS compared to those without and rose incrementally with the additional number of MetS components present (figure 5). The greatest increment in per patient costs was from no metabolic components present to one where there was a two-fold increase after which the costs increased by around 1.2 to 1.8 fold with each additional component.

### Sensitivity analysis

A number of sensitivity analyses were conducted (table 3). The total annual cost-of-illness of hypertension, with and without the presence of MetS did not vary significantly from the base case, with the exception of only including cases of incident or new onset diabetes rather than prevalent type 2 diabetes. In particular, in Germany by only including incident type 2 diabetes reduced the cost of hypertension with MetS from €24,427 million to €8,753 million. There was also a greater degree of variation in total costs when cardiovascular and type 2 diabetes costs were varied by plus or minus 20% than when drug costs and continuation rates were varied. We also assessed the impact of applying a discount rate of 3% to future costs. This would reduce the total annual cost of hypertension in 2020 from €42,769 million to €29,997 million in Germany; from €8,180 million to €5,737 million in Spain; and from €18,710 million to €13,123 million in Italy.

**Table 3:** Sensitivity analysis, total annual cost-of-illness (Euros, millions) of hypertension in the presence and absence of MetS; 2008

	Germany	Spain	Italy
**Baseline**			
Presence	24,427	1,908	4,877
Absence	5,341	2,683	5,956

Type 2 diabetes; including only incident type 2 diabetes			
Presence	8,753	1,050	1,775
Absence	3,382	2,115	3,876

Proportion treated; assume 58% hypertensive subjects were treated in Spain			
Presence	NA	2,010	NA
Absence	NA	3,058	NA

Continuation rate; vary by 20% above/below the mean			
+ 20% Presence	24,418	1,913	4,895
+ 20% Absence	5,357	2,707	6,035
- 20% Presence	24,406	1,898	4,850
- 20% Absence	5,302	2,635	5,831

Costs; vary by 20% above/below the mean *Drug costs*			
+ 20% Presence	24,553	1,928	4,929
+ 20% Absence	5,423	4,329	6,147
- 20% Presence	24,247	1,886	4,825
- 20% Absence	5,224	2,603	5,762
*CVD costs*			
+ 20% Presence	25,481	2,049	5,041
+ 20% Absence	5,682	2,934	6,276
- 20% Presence	23,373	1,769	4,714
- 20% Absence	5,000	2,431	5,637
*Type 2 diabetes costs*			
+ 20% Presence	27,743	2,102	5,573
+ 20% Absence	5,735	2,803	6,392
- 20% Presence	21,112	1,715	4,183
- 20% Absence	4,948	2,564	5,521

## Discussion

The results of this modelling study suggest that the presence of MetS in patients with hypertension significantly inflates the cost of illness due to the increase in cardiovascular events and cases of type 2 diabetes. These costs rise incrementally with the additional number of MetS components present. In Germany hypertensive patients with MetS account for over 60% of all hypertensive patients and contribute to 80% of the costs. In Spain and Italy they account for nearly a quarter of the hypertensive population and contribute to nearly half of the costs. The much higher prevalence in Germany can be explained by the country having one of the highest rates of abdominal obesity in Europe[47]. Mean annual costs per patient for those with MetS are two to three times higher than for those without. Antihypertensive drug costs make up less than 10% of overall costs of care in this high risk patient group with the management and treatment of cardiovascular events and type 2 diabetes accounting for the majority of costs.

These costs are set to rise in the future as the proportion of the population over the age of 50 years grows and the prevalence of the components of MetS increases. The most prevalent component of MetS in patients with hypertension is abdominal obesity which has reached epidemic proportions in Europe and currently shows no sign of slowing down[48].

This study used a prevalence-based modelling approach to estimate cost-of-illness and therefore is subject to a number of assumptions. Although efforts were made to ensure the best sources of data available to date were used in the model, as the published literature in this area is still relatively limited and national databases do not provide all appropriate data required to estimate epidemiological burden [49], there were a number of data gaps. As with all models that require some assumptions to be made an element of caution is required when interpreting the results. The assumptions used in this model are described in detail elsewhere[28]. For example we used a United States database to develop our conditional probability matrix which provided data on the probability of hypertensive subjects having no other MetS components, one, two, three or four and the probability of each combination occurring in each age and sex group. These probabilities were applied to country-specific prevalence data to break-down individuals into each risk group. We have no reason to assume that such conditional structures would be different between the U.S. and the three European countries as the clustering of individual MetS risk factors is unlikely to be significantly different.

Our estimates of the economic burden of MetS in patients with hypertension are on the conservative side for a number of reasons. First, no data were available to distribute costs of antihypertensive medications across risk groups and therefore costs have been distributed evenly across each of the five risk groups. Data suggest that the quantity and type of antihypertensive medication does differ between those with and without MetS. Those with MetS use significantly more ACE-inhibitors and ARBs and in general use antihypertensive drugs significantly more frequently than those without the syndrome[17,50]. A redistribution of costs accounting for this would not increase the overall drug costs, but would increase the proportion of drug costs that are attributable to MetS. Second, the number of cardiovascular events occurring in the population was predicted using data derived from a population initially free of cardiovascular disease (a proportion of the population had type 2 diabetes). The model therefore does not take into account the costs attributable to those hypertensive subjects with MetS and established cardiovascular disease, which would increase the total cost of illness. Finally, we only included healthcare costs in our model. Including costs relating to the loss in productivity due to morbidity or premature mortality would inflate cost-of-illness estimates by between 1.28 fold[51] and 10 fold[52]. Such wide estimates reflect the different methodologies used to calculate productivity losses.

Future prevalence estimates and costs are based on projected changes in the demographics of each country and the increase in prevalence of MetS components based on historical data trends. However, such forecasts do not take into account the potential impact of policy directives aimed at reducing the risk of developing one of the five MetS components. Future public health incentives will have an impact on future prevalence and costs however, these policies will be working against an increase in prevalence brought about by the aging population. The burden of illness in the future will therefore be dependent on how effective public health policies and guidelines are at preventing and treating MetS.

A handful of studies have assessed the prevalence of hypertension in patients with MetS, but none to date have assessed its economic impact. Previous population studies using ATP III criteria have shown that approximately 20% to 40% of the hypertensive population has MetS [20-23] giving rise to a prevalence in the general population of hypertension and MetS of 8% to 14% [20-22]. These estimates are similar to our estimates for Spain and Italy. Much higher estimates of the proportion of hypertensive subjects that have MetS are reported in primary care studies where the variation can be explained in part by the different sub-populations studied[13,17,24,25,27,53].

This study has highlighted the additional resource implications of hypertensive patients with MetS and the need to manage these patients effectively according to European guidelines to reduce the risk of cardiovascular disease and type 2 diabetes. However, a previous European study reported that fewer than 30% of treated hypertensive patients had their blood pressure controlled to levels recommended by European guidelines and that uncontrolled hypertension was strongly associated with MetS[27]. Not achieving recommended target levels of blood pressure control leaves these patients with elevated levels of risk. The study highlighted the importance of considering the patient's entire cardiometabolic profile when considering appropriate treatment rather than focusing solely on blood pressure targets alone[27].

European guidelines advocate that appropriate management of patients with hypertension should be based on their blood pressure level and overall cardiovascular risk profile. Guidelines recognise that due to the different mode of action of these classes, some drug groups are likely to offer greater benefits to different subgroups of patients [15,54,55]. In a recent reappraisal of guidelines on hypertension management, they suggest that drug choice should take into account contraindications as well as favourable effects in specific clinic settings. The guidelines do not recommend specific drugs for the treatment of hypertensive patients with metabolic syndrome, but do point out that '*there is no doubt that beta-blockers and diuretics (especially when combined together) have adverse metabolic effects and facilitate new onset diabetes in predisposed patients such as those with metabolic syndrome or impaired glucose tolerance*.' They go on to point out that there is still controversy over whether drug-induced new onset diabetes carries the same negative prognosis as naturally occurring diabetes [55]. In patients with diabetes the guidelines suggest that combination treatment is usually required and that '*a renin-angiotensin receptor blocker should always be included because of the evidence of its superior protective effect against initiation or progression of nephropathy*.'

Evidence suggests that newer antihypertensive medications are associated with a reduced risk of incident diabetes[39] and that they are also associated with better adherence to therapy[38,56,57]. Although meta-analyses suggest antihypertensive drugs have a similar effect on reducing cardiovascular events [58], there is some evidence to suggest that newer antihypertensive medications may lead to a greater reduction in the risk of first hypertension-related cardiovascular or diabetic event [57]. In addition, it has been recently demonstrated that obese hypertensive patients under drug-based weight loss therapy show significantly better weight reduction and improvement of insulin resistance when treated with newer antihypertensive medications compared to the older blood pressure lowering drugs (beta blocker, diuretics)[59]. Of the newer antihypertensive treatments ARBs have been found to be associated with the highest level of adherence[38,56,57] and the lowest association with incident diabetes[39]. Furthermore specific ARBs have also demonstrated favourable metabolic effects not present in other ARBs or ACE-inhibitors[60].

Following such guidelines which recommend the aggressive management of these high risk patients with a combination of lifestyle interventions to treat the MetS components present in the hypertensive individual and to prevent the onset of additional components is likely to lead to a significant reduction in costs of care. Newer antihypertensives lead to better control of blood pressure in part brought about by better adherence, thereby reducing the risk of cardiovascular disease. They also reduce the risk of new onset type 2 diabetes. Such outcomes are associated with significant associated costs. Therefore, in patients with hypertension and MetS, some of the drug costs of newer antihypertensive medications will be balanced by costs saved from reducing these negative outcomes. The magnitude of these savings is likely to depend on risk group. Patients with hypertension and four other components of MetS will demonstrate greater reductions in the incidence of cardiovascular events and new cases of type 2 diabetes and their associated costs as a result of treatment with newer antihypertensives compared to patients with hypertension only. Cost-effectiveness studies, assessing long-term costs and outcomes, will be required to demonstrate the incremental costs and benefits of new versus old antihypertensives for patients with hypertension and MetS. Studies have already demonstrated the cost-effectiveness of ARBs and ACE-inhibitors in patients at increased risk of diabetes and heart failure[54].

## Conclusion

The results of this study show that hypertensive patients with MetS are associated with higher costs of care compared to those without due to the increase in incident cardiovascular events and prevalent cases of type 2 diabetes. The resource implications of MetS rise linearly with each additional MetS component. This burden is forecast to rise in the future as a result of the aging population and an increase in the components of the syndrome. Prevention and appropriate treatment of MetS is likely to lead to a reduction in costs of care. Newer antihypertensive medications provide better control of blood pressure leading to reduced incidence of cardiovascular disease and type 2 diabetes. These benefits will be more marked in those hypertensive subjects who have a high number of MetS components clustered together. Following guidelines set out for this special group of patients, which advocate the use of newer antihypertensives, are likely to lead to longer-term economic benefits for the health service and quality of life improvement for patients.

## List of abbreviations

ACE: Angiotensin Converting Enzyme; ARB: Angiotensin II receptor blocker; ATP III: Adult Treatment Panel III of the United States National Cholesterol Education Program; CAGR: compound annual growth rate; HDL: high density lipoprotein; MetS: metabolic syndrome; NHANES: (United States) National Health and Nutritional Examination Survey.

## Competing interests

This project was funded by Bayer Schering Pharma AG.

## Authors' contributions

KU, JS, EA, CF, SL, WS and DJ were involved in conception and design. SL, WS and DJ were involved in the acquisition of data and analysis. KU, JS, EA and CF were involved in interpretation of data. SL, WS and DJ drafted the manuscript. KU, JS, EA and CF were involved in revising it critically for important intellectual content. All authors approved the final version to be published.

## Pre-publication history

The pre-publication history for this paper can be accessed here:

http://www.biomedcentral.com/1471-2458/10/529/prepub
